# Butyric acid increases transepithelial transport of ferulic acid through upregulation of the monocarboxylate transporters SLC16A1 (MCT1) and SLC16A3 (MCT4)

**DOI:** 10.1016/j.abb.2016.01.018

**Published:** 2016-06-01

**Authors:** Kerstin Ziegler, Asimina Kerimi, Laure Poquet, Gary Williamson

**Affiliations:** a School of Food Science and Nutrition, University of Leeds, Leeds LS2 9JT, UK; b Nestlé Research Center, CH-1000 Lausanne, Switzerland

**Keywords:** MCT, Butyric acid, Ferulic acid, Caco-2, Intestine, Short chain fatty acids, ABC, ATP-binding cassette, CT, monocarboxylate transporter, SCFA, short chain fatty acid, SMCT, sodium coupled monocarboxylate transporter, WGA, wheat germ agglutinin

## Abstract

Ferulic acid is released by microbial hydrolysis in the colon, where butyric acid, a major by-product of fermentation, constitutes the main energy source for colonic enterocytes. We investigated how varying concentrations of this short chain fatty acid may influence the absorption of the phenolic acid. Chronic treatment of Caco-2 cells with butyric acid resulted in increased mRNA and protein abundance of the monocarboxylate transporters SLC16A1 (MCT1) and SLC16A3 (MCT4), previously proposed to facilitate ferulic acid absorption in addition to passive diffusion. Short term incubation with butyric acid only led to upregulation of MCT4 while both conditions increased transepithelial transport of ferulic acid in the apical to basolateral, but not basolateral to apical, direction. Chronic treatment also elevated intracellular concentrations of ferulic acid, which in turn gave rise to increased concentrations of ferulic acid metabolites. Immunofluorescence staining of cells revealed uniform distribution of MCT1 protein in the cell membrane, whereas MCT4 was only detected in the lateral plasma membrane sections of Caco-2 cells. We therefore propose that MCT1 may be acting as an uptake transporter and MCT4 as an efflux system across the basolateral membrane for ferulic acid, and that this process is stimulated by butyric acid.

## Highlights

• Dietary fibre gives both short chain fatty acids and phenolic acids in the colon.
• Ferulic acid is partially taken into cells by the transporter MCT1.
• Ferulic acid is effluxed basolaterally by the transporter MCT4.
• Butyrate upregulates transporters of phenolic acids in a colonic model.
• A short chain fatty acid can regulate phenolic acid absorption.

## 1 Introduction

Fermentation of fibre by the gut microbiota results in the production of large quantities of short chain fatty acids (SCFA) amounting to millimolar concentrations in the colon [1], [2]. The SCFA butyric acid is taken up into colonic enterocytes either by passive diffusion of the free acid or via the apical uptake transporters monocarboxylate transporter 1 (MCT1) and sodium coupled monocarboxylate transporter 1 (SMCT1) [3], [4]. SCFA transport by facilitated diffusion is driven by one of three mechanisms; co-transport of protons, along the proton gradient created by the luminal pH of 6–7 (MCT1), transport of sodium ions along the sodium gradient maintained by basolateral sodium/potassium pumps (SMCT1), or through heteroexchange of monocarboxylates [5], [6]. After synthesis, the MCT1 transporter is assembled as a heterodimer with the chaperone peptide CD147 (basigin) which directs MCT1 insertion into the plasma membrane and the continued association of the two proteins is essential for correct functioning of the transporter [7]. No similar association between SMCT1 and a chaperone peptide has been reported [8].

The SCFAs taken up into the mucosal epithelium constitute the major energy source for cells of the large intestine [9], [10]. Butyric acid is reported to reduce the risk of colon cancer, protect the mucosa through stimulation of mucus secretion and tight junction integrity, and act as an anti-inflammatory agent [11], [12]. It also increases expression of MCT1 and its chaperone CD147 [13], [14], [15], [16], [17]. In the small intestine, low concentrations of SCFA correlate with lower MCT1 abundance [18] and in muscle cells, increasing lactate levels upregulated mitochondrial MCT1 expression as part of a lactate shuttle protein complex [19]. MCT1 is located on the vascular side of the epithelium, whereas MCT4 is both intracellular and lumen-facing, and so could transport SCFA into cells as well as shuttling SCFA metabolites intracellularly, while in goats, MCT4 was upregulated by long-term intra-ruminal infusion of sodium-butyrate [20].

Cereals and whole grain products are rich sources of phenolic compounds with ferulic acid being one of the most abundant [21]. Many phenolic acids are reported to act as anti-inflammatory agents and reduce the risk of Type 2 diabetes [22], [23], [24]. However only a very small percentage of ferulic acid is present in its free form as the majority is covalently linked to the carbohydrate matrix of the plant cell wall, in particular to side chains of the hemicellulose backbone [25] from which it is cleaved by intestinal esterases before absorption in the colon [26], [27].

Apart from their role as SCFA transporters, MCT1 and SMCT1 have also been suggested as the facilitated component of ferulic acid uptake [28], [29]. We therefore investigated whether there is a link between physiological butyric acid levels, as present in the colon, and the uptake of ferulic acid via MCT transport using the Caco-2 intestinal cell culture model.

## 2 Materials and methods

### 2.1 Materials

All cell culture reagents, acetonitrile, formic acid, 3,4-dimethoxycinnamic acid, ascorbic acid, dihydroferulic acid and ferulic acid were purchased from Sigma-Aldrich (St. Louis, USA). Cy3-conjugated donkey anti-mouse IgG and Alexa488-conjugated donkey anti-rat IgG were obtained from Jackson Immuno Research (West Grove, USA), fluorescein conjugated wheat germ agglutinin (WGA) from Vector laboratories (Burlingame, USA), ProLong Gold anti-fade reagent mounting medium from molecular probes (Life Technologies, Paisley, UK) and MCT monoclonal antibodies from Santa Cruz Biotechnology (sc-365501, sc-376139) and Insight Biotechnology (Middlesex, UK). Claudin monoclonal antibody (Claudin-1, #37-4900, Invitrogen) and all PCR consumables were from Life Technologies (Life Technologies, Paisley, UK). Ferulic acid sulfate was synthesized by Dr Nicolai U. Kraut (University of Leeds, Leeds, UK) and ferulic acid glucuronide was kindly provided by Prof. Denis Barron (Nestlé Institute of Health Sciences, Lausanne, Switzerland).

### 2.2 Cell culture

Caco-2 cells obtained from ATCC (LGC Standards, Middlesex, UK) were routinely cultured in 5 mM glucose Dulbecco's Modified Eagle's Medium (DMEM) supplemented with 15% Fetal Bovine Serum (FBS), 100 units/mL penicillin, 0.1 mg/mL streptomycin and 0.25 μg/mL Amphotericin B (full medium) at 37 °C with 5% CO_2_ in a humidified atmosphere. Cells were sub-cultured at ∼90% confluence and seeded on flasks at a density of 1 × 10^4^ cm^−2^. Cells were used between passage number 35 and 50.

### 2.3 Transport studies

Cells were seeded on 6-well Transwell plates (0.4 μM pore size, polycarbonate, Corning, UK) at a density of 6 × 10^4^ cm^−2^ and maintained for 22 days as above in full medium containing 10% FBS and either 250, 500 or 1000 μM butyric acid dissolved in ethanol (final concentration 0.5% for 250 and 1000 μM and 1% for 500 μM butyric acid) or the corresponding volume of ethanol for controls. The medium was changed every other day. For transport studies, cell monolayers were washed twice with Hank's Balanced Salt Solution (HBSS) and the Transepithelial Electrical resistance (TEER) of the cell layer was measured in HBSS containing 1.8 mM calcium chloride (+1.8 mM CaCl_2_). The average TEER value at day 22 was ∼400 Ω (with a growth area of 4.67 cm^2^). After TEER measurement, the buffer was replaced with 2 mL 500 μM ferulic acid dissolved in HBSS + 1.8 mM CaCl_2_ in the donor chamber and 2 mL of HBSS + 1.8 mM CaCl_2_ in the receiver chamber. The pH of both solutions was 7.4. For experiments investigating the impact of the MCT inhibitor phloretin, the compound was dissolved in DMSO and 300 μM was added to the ferulic acid containing transport solution. The final DMSO concentration was 0.1%; controls were supplemented with the corresponding volume of solvent. After 60 min incubation, 1.5 mL samples of the donor and receiver chambers were collected, acidified with 10 mM acetic acid and 100 μM ascorbic acid as antioxidant, 50 μM 3,4-dimethoxycinnamic acid as internal standard was added and the samples were analysed by LC-MS/MS. After sample collection, cells were washed three times with HBSS and lysed with 1000 μL of 80% methanol containing 100 μM ascorbic acid and 5 μM 3,4-dimethoxycinnamic acid. After vigorous mixing, cell lysates were centrifuged; the supernatant was dried under vacuum and reconstituted in 100 μL of 5% acetonitrile, 0.1% formic acid for chromatographic analysis (LC-MS/MS solvent A).

### 2.4 HPLC analysis

Aglycone analysis was done on an Agilent 1200 series HPLC equipped with a diode array detector (DAD). Separation was achieved on an Agilent ZORBAX Eclipse Plus C18 column (2.1 × 100 mm, 1.8 μm). Solvent A consisted of 94.9% MilliQ water, 5% acetonitrile and 0.1% formic acid and solvent B was 94.9% acetonitrile, 5% MilliQ water and 0.1% formic acid. The solvent gradient profile was as follows: 0–12 min: linear gradient from 0% to 50% solvent B; 12–18 min: linear gradient from 50% to 100% solvent B; 18–20 min: 100% solvent B; 20–24 min: linear gradient from 100% to 0% solvent B; 24–31 min: 0% solvent B at 0.2 mL/min. The peak areas determined at 320 nm were utilised to quantify both ferulic acid and the internal standard 3,4-dimethoxy-cinnamic acid by comparison to original standards.

### 2.5 LC-MS/MS analysis

Samples were analysed for ferulic acid metabolites on an Agilent 6410 LC-MS/MS as described previously [30] with the following modifications: solvent A consisted of 95% deionised water, 5% acetonitrile and 0.5% formic acid, solvent B consisted of 95% acetonitrile, 5% deionised water and 0.5% formic acid. A solvent gradient was run with the following profile: 0% solvent B from 0 to 3 min, a linear increase from 5% to 30% solvent B from 3 min to 15 min, 100% solvent B from 15.1 min to 17.5 min and 0% solvent B from 17.6 min to 21.5 min. Analytes were identified and quantified using original standards based on the following MRM pairs:

*m*/*z* = 369 → 113 for ferulic acid glucuronide,
*m*/*z* = 273 → 178 for ferulic acid sulfate,
*m*/*z* = 195 → 121 for dihydroferulic acid,
*m*/*z* = 193 → 178 for ferulic acid and
*m*/*z* = 207 → 103 for the internal standard 3,4-dimethoxycinnamic acid.

For results shown in Fig. 6B, C and D, the concentrations were adjusted to the original volume of the corresponding compartment. The volume of a cell monolayer was estimated by immunofluorescence staining of the cell membrane. The distance between the apical and basolateral membrane was estimated by confocal microscopy and was an average of 15 μm from 13 independent experiments. With a growth area of 4.67 cm^2^ for Transwell inserts, the volume of a cell monolayer was calculated to be 7 μL. As cell lysates were reconstituted in 100 μL of solvent A, a 14.3× dilution is taken into account. For intracellular ferulic acid metabolites the concentration obtained by LC-MS/MS analysis was multiplied by this dilution factor. Samples collected from the apical or basolateral well were injected without any further processing.

### 2.6 Gene expression

Cells were seeded on solid well plates and maintained as for transport experiments. For gene expression studies, the culture medium was replaced with medium containing 1000 μM butyric acid dissolved in ethanol (final concentration 0.5%) or the corresponding volume of ethanol for controls. Cells were either incubated with butyric acid for their entire differentiation time of 22 days (chronic) or for 24 h (acute) starting at day 21. Cells were then washed with ice cold PBS, scraped and mRNA was extracted using the Ambion RNAqueous kit (AM1912, Ambion, Life Technologies, USA), according to the manufacturer's protocol. RNA was transcribed to cDNA using the Applied Biosystems high capacity RNA to cDNA kit (4387406, Life Technologies, USA) and gene expression was determined by real-time PCR using the Applied Biosystems TaqMan gene expression assay and gene expression master mix (4369016, Life Technologies, Foster City, California, USA) according to the manufacturer's protocol on a StepOnePlus real-time PCR system (Applied Biosystems, USA). The Applied Biosystems ID of the primer/probe set for *SLC16A1* was Hs00161826_m1, for *SLC16A3* it was Hs00358829_m1, for *SLC16A4* it was Hs01006127_m1, the probe number for *SMCT1* was Hs00377618_m1, for *ABCB1* Hs00184500_m1, for *ABCC2* Hs00166123_m1, for *ABCG2* Hs01053790_m1 and NM_002046.3 for the reference gene GAPDH. Expression of the gene of interest is presented normalised to the reference gene GAPDH (multiplexed) and to the expression in untreated control cells using the ΔΔCt method.

### 2.7 Immunofluorescence staining

Caco-2 cells were seeded on Millicell cell culture inserts (12-well, PET 0.4 μm pore size, Millipore) at a density of 6 × 10^4^ cm^−2^ and maintained for 21 days. For staining, cells were fixed with 4% *para*-formaldehyde in PBS and incubated with WGA for 10 min. Following permeabilisation with 0.1% Triton-X100 cells were incubated either with MCT1 or MCT4 antibody at a dilution of 1:50 for 1 h at room temperature. After washing with PBS, cells were further incubated with Cy3-conjugated donkey anti-mouse IgG at a 1:300 dilution. Cell layers were stained with 0.2 mg/mL DAPI and mounted on microscopy slides with ProLong Gold antifade reagent mounting medium and imaged using a Zeiss LSM510 confocal microscope.

### 2.8 Protein analysis

For protein detection, Caco-2 cells grown on Transwell plates as before were washed with ice cold PBS, scraped and lysed in Bicine-Chaps buffer (ProteinSimple) containing 1% protease inhibitor cocktail. The lysate was centrifuged at 14,000× *g* for 10 min and the total protein concentration of the supernatant was determined by BCA microplate assay according to the manufacturer's instructions (ThermoFisher Scientific, UK). For analysis of MCT1 and MCT4 protein, the ProteinSimple ‘WES’ system was used. Samples were denatured by incubation with 0.1× sample buffer (ProteinSimple) at 37 °C for 20 min. Claudin1 (1:50) was used as a loading control and run in the same capillary as MCT1 (1:25) or MCT4 (1:25) following optimisation (Fig. 4). The primary antibody incubation was 1 h. A standard curve was constructed with different amounts of Caco-2 cell lysate to test linearity of both antibodies and a loading sample concentration of 0.5 mg/ml was used as found optimal for signals of all antibodies (Fig. 4).

### 2.9 Statistics

All values shown are the mean of three to six independent experiments ± standard error of the mean, as indicated in the figure legends. For analysis of statistical significance, independent samples Student's t-test was employed and analysis carried out by SPSS Statistics (v22, IBM).

## 3 Results

**Fig. 1:**
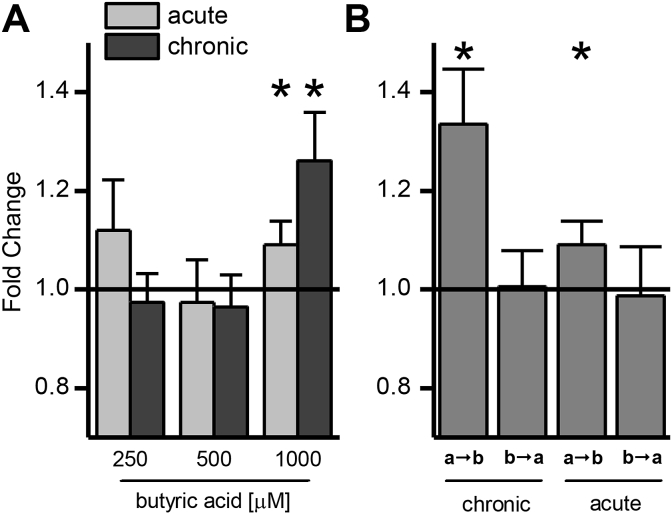
Impact of butyric acid on ferulic acid transport across differentiated Caco-2 cell monolayers. (A) Impact of incubation time and concentration of butyric acid compared with vehicle-treated controls. Cells were either incubated for their entire differentiation time of 22 days (chronic) or for 24 h starting at day 21 after seeding (acute). Butyric acid was dissolved in ethanol (final concentration 0.5% for 250 and 1000 μM and 1% for 500 μM butyric acid). Controls were incubated with the equivalent amount of ethanol for the corresponding time. (B) Direction specific effect of 1000 μM butyric acid on transport of ferulic acid. (n = 6; * = p ≤ 0.05).

**Fig. 2:**
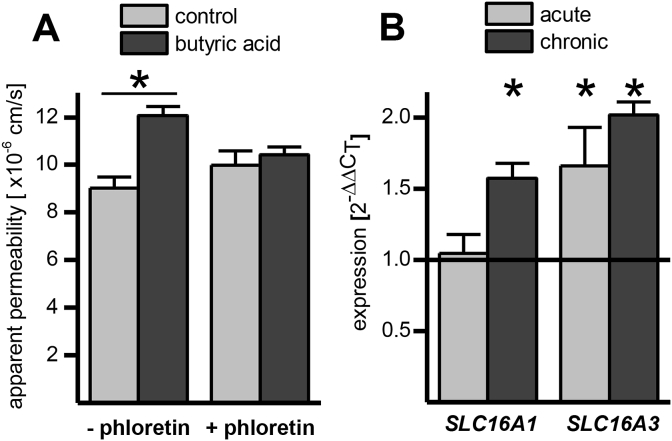
(A) Impact of phloretin (300 μM) on apical to basolateral transport of ferulic acid across Caco-2 monolayers chronically supplemented with butyric acid (1000 μM). (B) Impact of chronic and acute butyric acid supplementation on SLC16A1 (MCT1) and SLC16A3 (MCT4) mRNA (n = 6; * = p ≤ 0.05).

**Fig. 3:**
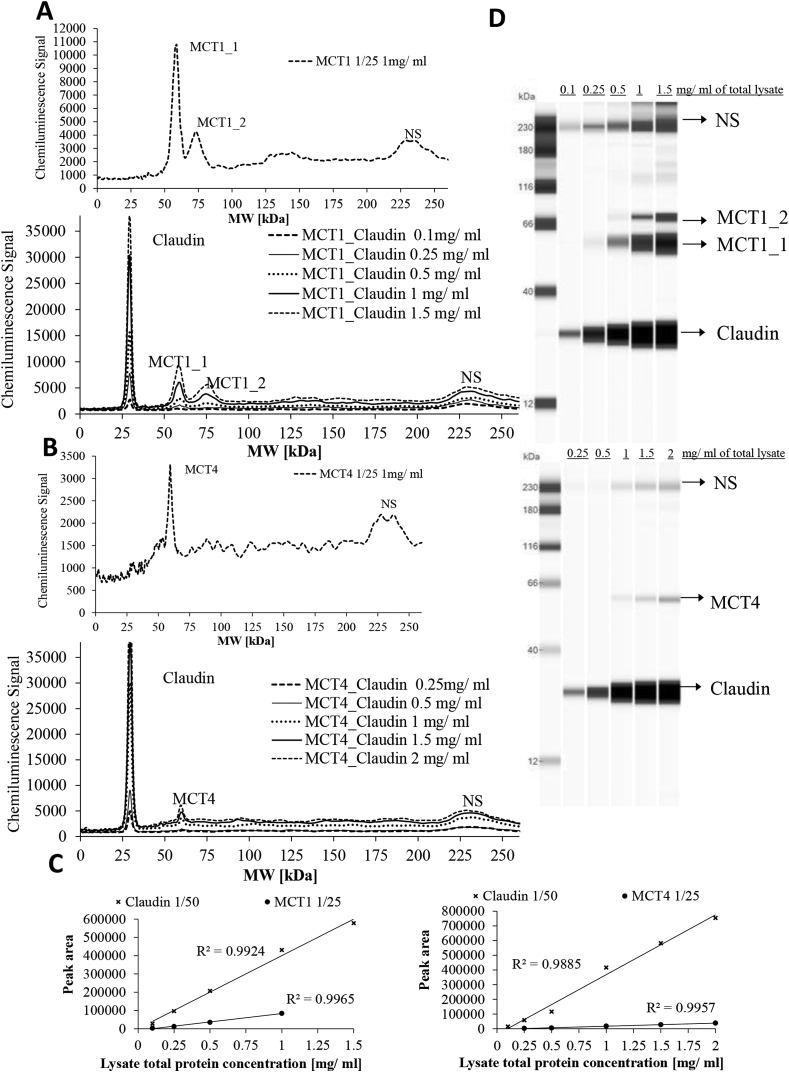
Antibody validation for protein analysis of MCT1 and MCT4 by ProteinSimple WES system. (A) Pherogram view of 1 mg/ml of Caco-2 whole cell lysate analysed for MCT1 alone and of various amounts of Caco-2 whole cell lysate analysed for MCT1 when run with claudin in the same capillary for standard curve construction. (B) Pherogram view of 1 mg/ml of Caco2 whole cell lysate analysed for MCT4 alone and of various amounts of Caco-2 whole cell lysate analysed for MCT4 when run with claudin-1 in the same capillary for standard curve construction. (C) Standard curve for MCT1 and claudin-1 run in the same capillary and MCT4 and claudin-1 run in the same capillary in Caco-2 whole cell lysate. (D) Lane view of different amounts of whole cell lysate analysed for standard curve construction. NS denotes non-specific interactions of the MCT1, MCT4 antibodies with the fluorescence standards used in the ProteinSimple WES system.

**Fig. 4:**
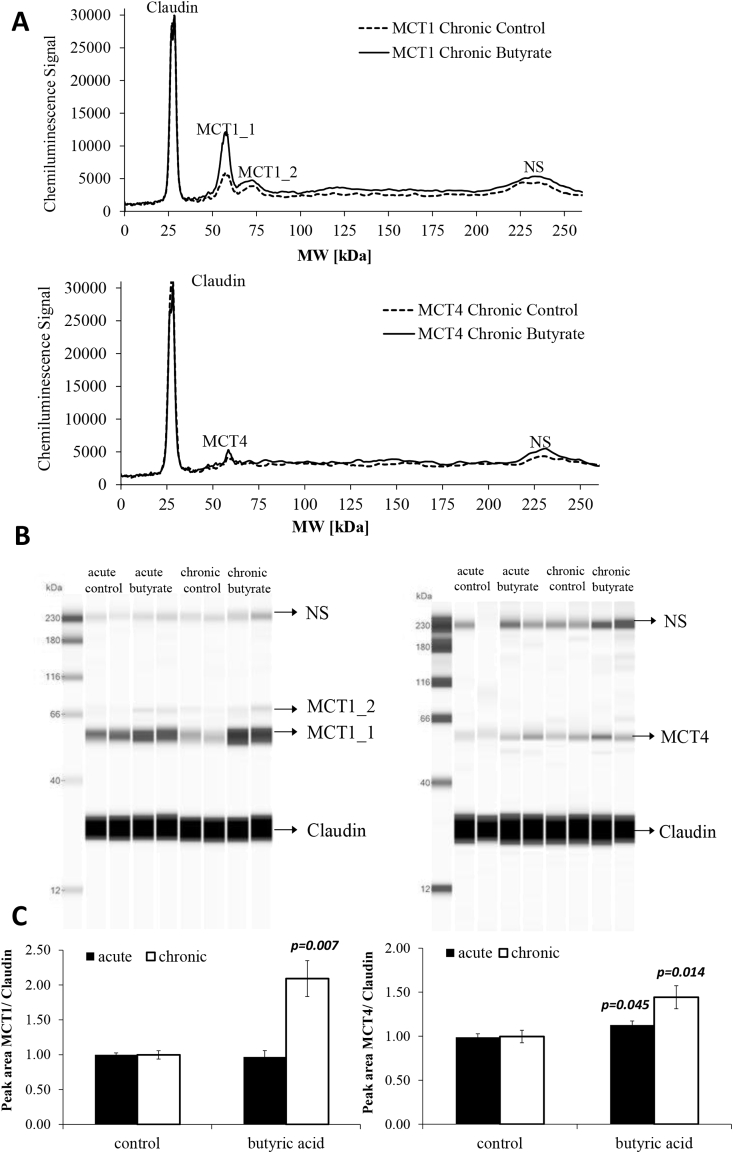
Effect of acute and chronic butyrate treatment on MCT1 and MCT4 protein levels in Caco-2 cells detected with the ProteinSimple WES system. (A) Pherogram view of a representative biological replicate for control and chronic treated butyrate samples analysed for MCT1 and MCT4. (B) Lane view of two representative biological replicates for control and butyrate acute and chronic treated samples analysed for MCT1 and MCT4. (C) Peak areas of MCT1 and MCT4 protein levels normalised to claudin-1, detected in 0.5 mg/ml Caco-2 whole lysate after acute or chronic treatment with 1 mM butyrate and controls treated with vehicle only; n = 6, error bars represent standard error of the mean.

The impact of increasing concentrations of butyric acid on ferulic acid transport was investigated using the intestinal Caco-2 cell culture model. Only the highest concentration of 1000 μM butyrate was able to significantly modulate ferulic acid uptake, and only in the apical to basolateral direction. Chronic butyric acid supplementation had a much greater effect than acute treatment (Fig. 1A). A directional change in transport as shown in Fig. 1B argues against a change in passive diffusion and suggests that transporters are involved, possibly MCTs, as previously hypothesised [3], [4]. In addition, the presence of 300 μM phloretin (an MCT inhibitor) during the transport abolished the effect of butyric acid pre-treatment, indicating that an MCT member is involved in the increase of ferulic acid transport (Fig. 2A). MCT1 mRNA (Fig. 2B) and protein levels (Fig. 3, Fig. 4) were higher following chronic but not acute treatment, whereas for MCT4 changes were significant after both treatments; MCT5 mRNA was not affected, and no SMCT mRNA was detected by droplet digital PCR analysis (ddPCR, Bio-Rad, Hemel Hempstead, UK, data not shown).

**Fig. 5:**
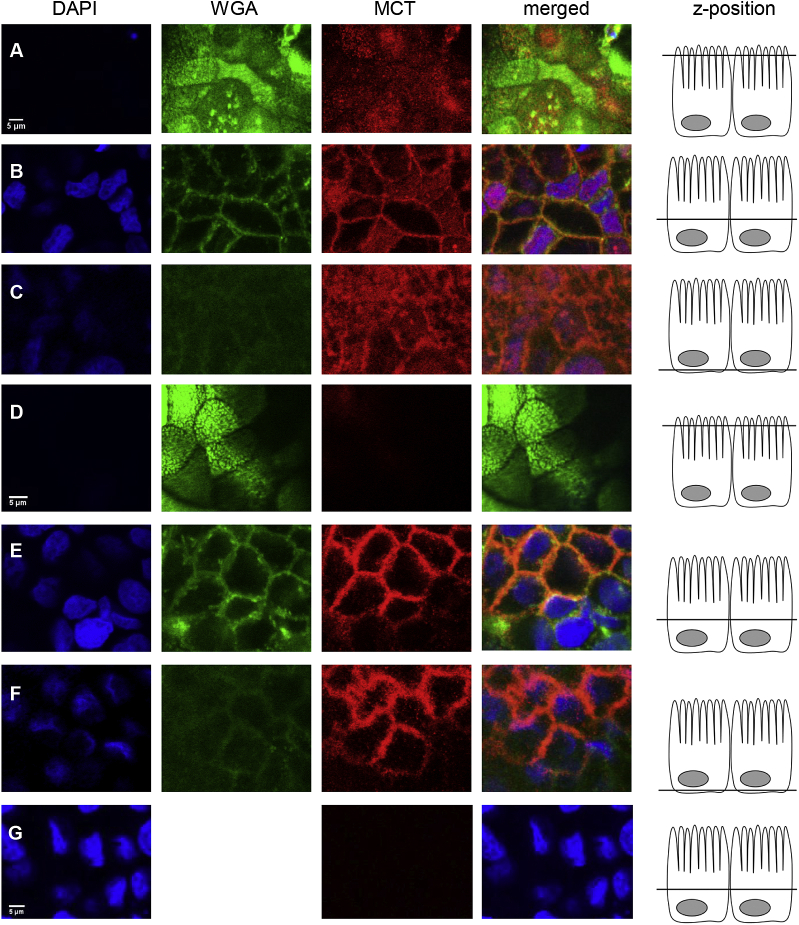
Indirect immunofluorescence detection of MCT1 and MCT4 in differentiated Caco-2 monolayers. Cells were incubated with DAPI, membrane marker wheat germ agglutinin (WGA) and either mouse anti-hMCT1 (rows A to C) or mouse anti-hMCT4 (rows D to F) primary antibody and Cy3-conjugated donkey anti-mouse secondary antibody. MCT is shown in red, appearing orange when co-localising with WGA shown in green and appearing purple when co-localising with DAPI shown in blue. Row G shows control cell layers incubated with DAPI and secondary antibody only. Scale bars (5 μm) are shown in the lower left corner of DAPI images. Scale bar in row A applies to all images in row A, B and C, scale bar in row D applies to all images in row D, E and F and scale bar in row G applies to row G only (n = 3).

To further elucidate the role of MCT1 and 4, indirect immunofluorescence staining of Caco-2 cell monolayers was performed (Fig. 5). MCT1 protein (in red) was detected in the plasma membrane on the apical, lateral and basal side, co-localising with the plasma membrane marker, WGA (shown in green, appearing orange in the merged fluorescence images when co-localising with red MCT1). MCT1 was also detected in the nuclear envelope, co-localising with DAPI (shown in blue, appearing purple in merged images when co-localising with red MCT1). MCT4 was mainly localised in the lateral plasma membrane with only very low signal originating from the apical or basal membrane. No intracellular signal was detected.

**Fig. 6:**
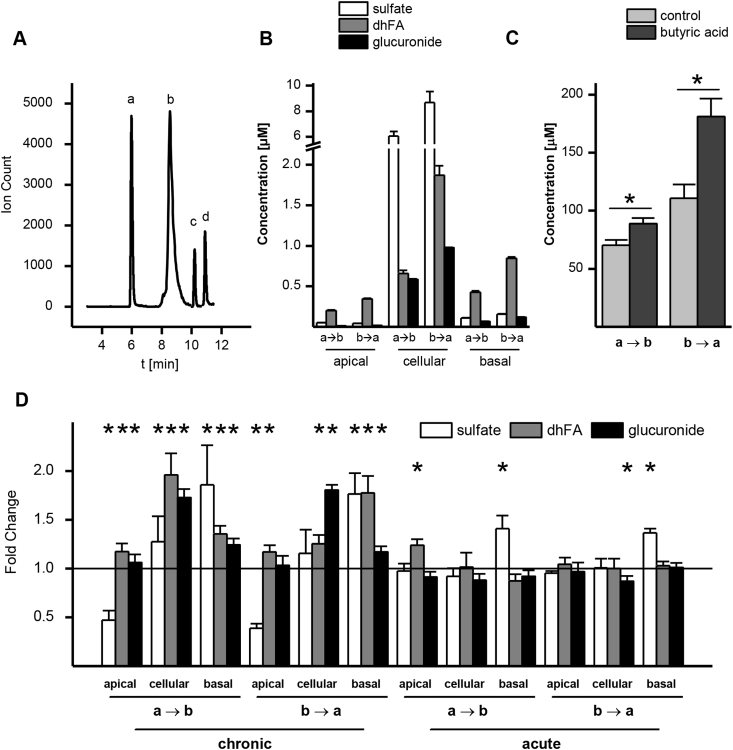
Ferulic acid metabolism by Caco-2 cells. (A) LC-MS/MS chromatogram of equimolar concentrations of analytes (1000 μM). a = ferulic acid glucuronide (*m*/*z* = 369 → 113), b = ferulic acid sulfate (*m*/*z* = 273 → 178), c = dihydroferulic acid (*m*/*z* = 195 → 121), d = ferulic acid (*m*/*z* = 193 → 178). (B) Concentration of metabolites detected in apical, basolateral and cell lysate samples after ferulic acid (500 μM) transport in uptake (a → b) or efflux (b → a) direction. (C) Intracellular ferulic acid concentrations after 1 h uptake or efflux transport across cells chronically treated with butyric acid (1000 μM). (D) Change in metabolite production after acute or chronic butyric acid (1000 μM) treatment of Caco-2 cells, expressed in fold changes compared with control condition without butyric acid (n = 6; * = p ≤ 0.05).

In addition to the transport of ferulic acid, the impact of chronic and acute butyric acid on ferulic acid metabolism in Caco-2 cells was investigated. Fig. 6 shows concentrations of the three major ferulic acid metabolites (ferulic acid sulfate, dihydroferulic acid and ferulic acid glucuronide) for both uptake (a → b) and efflux (b → a) transport. Concentrations were adjusted for the original volume of the sample as stated in the experimental section. All metabolites were more abundant in the basolateral well compared to the apical well, but the highest concentrations were found intracellularly. Dihydroferulic acid was the predominant metabolite in the donor and acceptor wells while ferulic acid sulfate was the principal metabolite intracellularly, reaching concentrations of up to 9 μM. Metabolite concentrations were higher when ferulic acid was applied to the basolateral side (b → a) than the apical (a → b), and this difference could be explained by the increase in the intracellular concentration of the ferulic acid which was further amplified by the butyric acid supplementation (Fig. 6C).

**Fig. 7:**
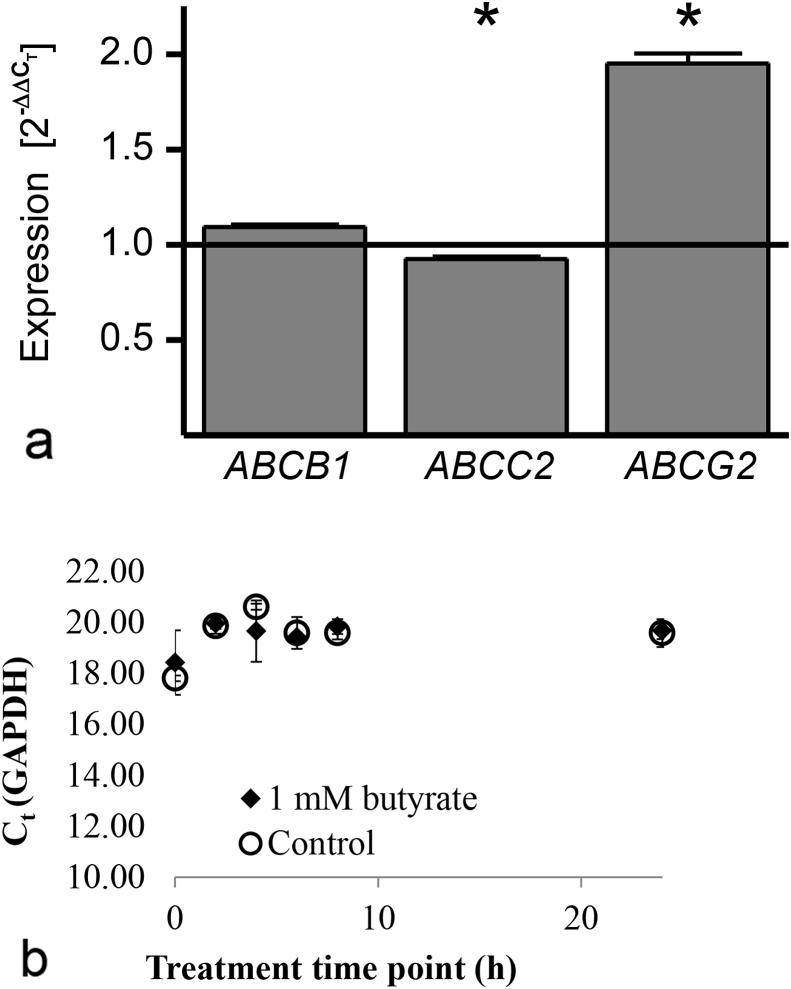
A. Expression of apical ABC-efflux transporters in Caco-2 cells after 22 day treatment with 1000 μM butyric acid. (n = 5, * = p ≤ 0.05). Expression is relative to GAPDH. B. GAPDH expression. There were no differences in GAPDH expression between 1 mM butyrate (C4) and control treatment for up to 24 h starting at day 20 after seeding. There were also no changes when cells were incubated for their entire differentiation time of 21 days either when the cells were grown on transwell plates or solid support dishes: C_t_ values in solid wells: starting (24 h) control, 16.68 ± 0.15; butyrate, 16.78 ± 0.19; after 21 days, control 18.38 ± 0.21; butyrate, 18.48 ± 0.30. C_t_ values in permeable transwells: starting (24 h) control, 19.59 ± 0.54; butyrate, 19.69 ± 0.34; after 21 days, control 17.74 ± 0.14; butyrate, 17.96 ± 0.23 (n ≥ 4).

Chronic butyric acid supplementation had a pronounced impact on metabolism when compared to the acute treatment (Fig. 6D). Acute supplementation increased ferulic acid sulfate levels on the basolateral side and dihydroferulic acid on the apical side when ferulic acid was applied apically, and decreased intracellular levels of ferulic acid glucuronide when applied basolaterally. With chronic butyric acid supplementation, levels of all metabolites increased, except for apical levels of ferulic acid sulfate. To investigate whether these changes in ferulic acid metabolites could be linked to effects on apical efflux transporters following the butyric acid treatment, mRNA levels of the most abundant apical ATP-binding cassette transporters in Caco-2 cells, ABCB1, ABCC2 and ABCG2, were measured (Fig. 7). Gene expression of ABCG2 was significantly increased after chronic butyric acid treatment, whereas ABCC2 expression was marginally but significantly reduced.

## 4 Discussion

MCT1 acts either as a symporter, facilitating proton coupled transport of monocarboxylates, or as an antiporter exchanging one monocarboxylate against another without proton movement [7]. MCT1 expression increases in the presence of butyric acid, a microbial metabolite reaching high concentrations of up to ∼25 mM in the colon [2]. In the current study, pre-treatment of intestinal Caco-2 cells with 1000 μM butyric acid resulted in increased transport of ferulic acid in the uptake direction, an effect that was abolished by the MCT inhibitor phloretin. Ferulic acid is a potential allocrite of MCT1 and, in the current study, upregulation of MCT1 by chronic butyric acid supplementation correlated with an increase in ferulic acid uptake into the cell. However, ferulic acid transport across the cell monolayer was increased even when MCT1 expression was not, suggesting that other MCT members may be involved. We found that MCT4 was also affected by butyric acid treatment as noted before [20]. MCT4 is highly expressed in the colon and in Caco-2 cells and also reported to be inhibited by phloretin [3], [31], [32]. Immunofluorescence staining of Caco-2 cells revealed the presence of MCT4 solely in the basolateral plasma membrane whereas MCT1 was more uniformly distributed.

**Fig. 8:**
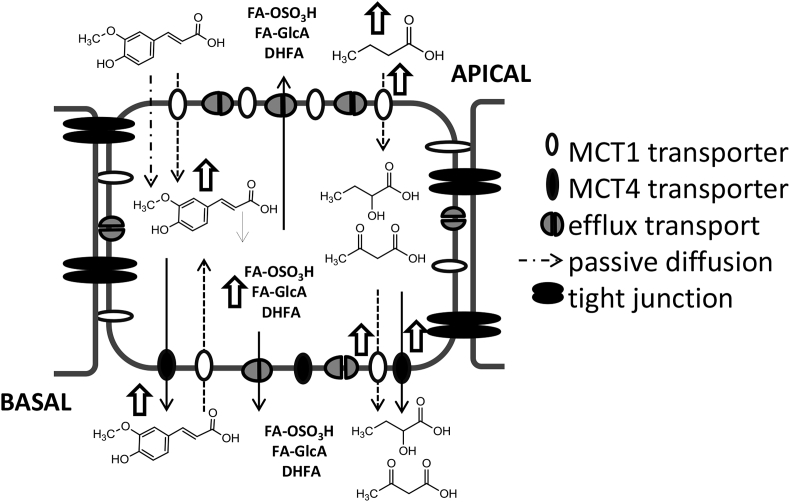
Proposed mechanism of ferulic acid transport across Caco-2 monolayers. Metabolites of ferulic acid: FA-OSO_3_H; ferulic acid sulfate, FA-GlcA; ferulic acid glucuronide, DHFA; Dihydroferulic acid. Butyrate is used mainly as an energy source for colonocytes, and selected metabolites are shown for illustrative purposes. Open arrows indicate and increase in the adjacent transporter or metabolite.

Taken together, these results suggest (Fig. 8) that ferulic acid, applied to either the apical or basolateral side, is taken up into the cell by MCT1, which is more abundant after chronic butyric acid treatment, resulting in elevated intracellular concentrations. In agreement, after acute butyric acid treatment, no increase in intracellular concentrations was observed as MCT1 levels were not changed. Hence we hypothesise that MCT1, although also localised at the apical side, is only able to import ferulic acid into the cell, as there is no increase in ferulic acid transport to the apical side after chronic butyric acid supplementation. MCT4, on the other hand, seems to only transport ferulic acid from the intracellular environment to the basolateral side and not in the reverse direction, as intracellular ferulic acid concentrations remained stable when ferulic acid was applied to the basolateral side following acute butyric acid treatment that increased MCT4 levels. The lack of MCT4 in the apical membrane has the consequence that only apical to basolateral transport is affected by butyric acid and not basolateral to apical transport. However the observation that MCT1 only imports and MCT4 only exports ferulic acid contradicts the idea that both transporters can translocate monocarboxylates in and out of the cell, only depending on the concentration gradient of the allocrite [33]. This could be explained by the fact that bidirectional transport by MCT1 and MCT4 has mostly been studied in non-differentiated cell lines and oocytes using small endogenous compounds such as lactic acid, which may follow different transport kinetics compared to larger molecules such as ferulic acid. The directionality of transport by MCTs in Caco-2 cells has also been observed previously; the transport of salicylic acid, another substrate of MCT1 could be inhibited by phloretin in the apical to basolateral but not in the basolateral to apical direction [34].

Findings from transport experiments are consistent with changes in metabolism of ferulic acid, after chronic or acute butyric acid treatment. MCT1 changes after chronic butyric acid-treatment resulted in increased intracellular ferulic acid concentrations and a higher rate of formation of ferulic acid metabolites due to substrate availability. Similarly higher metabolite concentrations were observed after efflux (b → a) compared to uptake (a → b) transport with the only exception being ferulic acid sulfate which decreased on the apical side with butyric acid supplementation. This could be due to decreased expression of the apical efflux transporter ABCC2, implying that ferulic acid sulfate may be a substrate for ABCC2. ABCG2 in comparison was strongly upregulated with butyric acid treatment and may have contributed to increased efflux of ferulic acid glucuronides or dihydroferulic acid to the apical side.

There are conflicting reports regarding the intestinal localisation of MCT1; detected in only the apical membrane of Caco-2 cells [35] and of enterocytes in the human large intestine [31]; detected in only the basolateral [36] membrane of enterocytes in the human large intestine; detected in the apical membrane of colonocytes from pig [37] and rat [17], but in the basolateral membrane of colonocytes from hamster [38] and cow [39]. MCT4 has been detected in the basolateral membrane of human mucosal cells [31] but its localisation was found to vary in the ruminant intestine. In the small intestine MCT4 was detected with high intensity in the apical and basolateral membranes at the villi tip but basolateral localisation decreased down the crypt-villus axis with MCT4 located mainly in the apical membrane of crypt cells. In the large bovine intestine MCT4 distribution was converse, with apical localisation in villi tips and basolateral localisation in crypts [40]. A possible explanation for this variation in reports for MCT1 localisation in different tissues and cell lines was recently suggested. Compared to MCT4, which contains a strong C-terminal sorting signal targeting the protein to the basolateral membrane of epithelial cells, MCT1 does not contain any sorting signal but is localised by its chaperone CD147, which bears a signal sequence for basolateral sorting, as well as a weak apical targeting signal. Through the tight association between MCT1 and CD147, MCT1 is additionally targeted to the basolateral membrane in some types of cells and solely to the apical side in other types such as the retinal pigment epithelium when the basolateral targeting sequence is not recognised. CD147 knock down studies have shown that in the absence of the chaperone, MCT1 and MCT4 proteins are not detected in the plasma membrane, but instead accumulate in the Golgi [41], [42]. CD147 is highly glycosylated which may also play a role in its apical or basolateral targeting [43], [44], [45].

SMCT1 has also been proposed to facilitate ferulic acid transport [46] and its presence has been reported in Caco-2 cells [15]. However we could not detect any SMCT1 mRNA copies even by ddPCR. The gene expression of drug transporters and metabolizing enzymes in Caco-2 cells varies substantially between laboratories [47], sometimes depending on the cell line origin and culturing conditions [48]. The absence of SMCT1 in the Caco-2 cells used in the experiments described here could be consistent with reports of SMCT1 down-regulation in other colon cancer cell lines. Exon 1 of SMCT1 was found to be hypermethylated and therefore not expressed in over 50% of primary colon cancers and adenomas and in some colon cancer cell lines [49], [50].

We conclude a moderate concentration of butyric acid is able to modulate MCT1 and MCT4 levels, resulting in increased uptake of ferulic acid into colonocytes and basolateral transport. Our findings could potentially translate to higher concentrations of ferulic acid in systemic circulation *in vivo*.

## Authorship contributions

Participated in research design: Ziegler, Kerimi, Poquet and Williamson.

Conducted experiments: Ziegler, Kerimi.

Performed data analysis: Ziegler, Kerimi.

Wrote or contributed to the writing of the manuscript: Ziegler, Kerimi, Poquet and Williamson.

## Conflict of interest

This work received partial funding from Nestle, and LP is an employee of Nestle Research Center, Switzerland; GW has recently received other research funding from Florida Department of Citrus, and conducted consultancy for Nutrilite, USA.
